# Randomized crossover trial on motor and non-motor outcome of directional deep brain stimulation in Parkinson’s disease

**DOI:** 10.1038/s41531-024-00812-0

**Published:** 2024-10-26

**Authors:** Alireza Gharabaghi, Idil Cebi, Dallas Leavitt, Maximilian Scherer, Patrick Bookjans, Bastian Brunnett, Luka Milosevic, Daniel Weiss

**Affiliations:** 1 Institute for Neuromodulation and Neurotechnology, University Hospital and University of Tübingen, Tübingen, Germany; 2 Center for Bionic Intelligence Tübingen Stuttgart (BITS), Tübingen, Germany; 3 German Center for Mental Health (DZPG), Tübingen, Germany; 4 Center for Neurology, Department for Neurodegenerative Diseases, and Hertie Institute for Clinical Brain Research, University Tübingen, Tübingen, Germany; 5 Max-Planck-Institute for Biological Cybernetics, Tübingen, Germany; 6 Clinical and Computational Neuroscience, Krembil Research Institute, University Health Network, Toronto, ON Canada

**Keywords:** Parkinson's disease, Randomized controlled trials

## Abstract

Deep brain stimulation (DBS) with electric field steering may avoid areas responsible for side effects. This prospective randomized cross-over trial compared omnidirectional (OS) and directional (DS) subthalamic DBS in 19 patients. Electromyographically measured rigidity was the primary outcome. Motor and non-motor scores were secondary outcomes. There were no significant differences between OS and DS. In the acute setting, both conditions improved motor scores compared to no stimulation. Motor symptoms improved after 3 weeks of OS relative to acute measurements, whereas they worsened under DS. The more ventral the active contact, and the less the motor improvement sweet spot was stimulated, the greater the benefit of DS over OS for executive function. Accurate OS of the dorsal subthalamic nucleus ensures motor and non-motor improvements. While DS can mitigate executive decline stemming from off-target stimulation, it may lead to worse motor outcomes. Larger, long-term studies are needed to confirm these findings. (Registration: subthalamic steering for therapy optimization in Parkinson’s Disease ClinicalTrials.gov: NCT03548506, 2018-06-06).

## Introduction

Deep brain stimulation (DBS) of the subthalamic nucleus (STN) is a proven and cost-effective therapy for Parkinson’s disease (PD), offering superior motor, non-motor, and quality of life outcomes compared to best medical therapy once dopaminergic response fluctuations occur^1–7^. The greatest efficacy of DBS is often achieved in the motor portion of the STN and its immediate vicinity^8^. However, the spread of stimulation current to nearby structures can lead to side effects such as muscle contractions, paresthesia, postural instability, speech impairment, reduced verbal fluency, involuntary eye movements, and cognitive impairment^9–11^. Motor side effects are usually recognized and managed quickly and may require lead revision surgery if they occur at subtherapeutic stimulation intensities^12^, whereas non-motor side effects such as cognitive impairment often go unnoticed during acute parameter optimization.

Technological advances, particularly new electrodes that divide the middle two of the usual four-ring contacts into three segments, allow for directional (DS) in addition to circular/omnidirectional (OS) stimulation. Previous studies, both intraoperative^13,14^ and extra-operative^7,15–17^, report an increased therapeutic window for DS vs. OS, suggesting that DS may avoid structures that cause side effects, although long-term relevance, such as compensating for suboptimal electrode placement and reducing revision surgery, remains uncertain.

Despite advances in image-guided neurosurgical targeting^18,19^, refined intraoperative techniques^20,21^, awake procedures^22^, and novel stimulation paradigms^23^, side effects and insufficient benefits lead to revision surgery in 15–30% of patients^12^. A simulation study indicates that DS reduces off-target stimulation but struggles with electrode displacements >1 mm^24^. Additionally, 6 of 42 patients in another study required surgical revision despite the ability to reduce side effects with DS, suggesting that improvements in surrogate parameters like the therapeutic window do not always translate into clinical benefits^25,26^.

On this basis, it is of particular relevance to determine the potential of DS to improve the therapeutic precision in the majority of patients with well-placed electrode leads where beneficial therapeutic effects can be achieved without acute side effects. For these cases, it is unknown as to whether DS allows for a more efficient stimulation of the target structure, e.g., by lowering the stimulation intensity required to achieve the desired clinical benefit, also for longer observation periods beyond acute assessments. Such long-term assessments would allow to estimate potential benefits of DS also with regard to non-motor side effects, e.g., cognitive impairment.

In this context, an important consideration is that therapeutic impedance and surface current density are dependent on the electrode surface, and systematically differ between larger ring and smaller segmented contact sizes; associated with greater current density of the latter^27^. It has therefore been suggested that stimulation intensity adjustments using DS should be performed using smaller amplitude increments (i.e., 0.1–0.3 mA compared to the traditional 0.5 mA). Moreover, it needs to be considered that because of the higher impedance (1.8–2.2 times that of standard ring contacts) due to the smaller stimulating surface of segmented contacts, stimulation at a particular intensity is associated with a greater total electrical energy delivered (TEED) when using DS compared to OS. As such, these settings would impose a greater energy burden and quicken battery consumption; which is an important factor influencing the frequency of subsequent surgical procedures for battery replacement. Already an 18% increase from the average TEED with DS vs. OS, would lead to as much as a year of battery lifespan lost. Therefore, the desired stimulation effects need to be achieved with at least the same TEED, i.e., about 30% lower stimulation amplitudes of DS vs. OS, to avoid a negative influence on the battery lifespan^28^. These aspects need to be considered when aiming for overall improvements of clinical care. Moreover, the stimulation effects in previous studies were only recorded clinically using an ordinal scale (i.e., the Unified Parkinson’s Disease Ratings Scale; UPDRS) and were therefore dependent on subjective assessments of the symptoms.

Considering these factors, we designed a study to systematically compare the efficacy of DS vs. OS in a prospective, randomized, double-blind, cross-over clinical trial. We used electromyography (EMG) to assess muscle rigidity (primary outcome) and clinical measures (secondary outcome), performed stimulation titration in small increments (0.2 mA steps) for both conditions, maintained TEED between DS and OS, used randomized, double-blind evaluations after both acute and chronic periods, and assessed both motor and non-motor symptoms. In addition, ancillary analyses explored whether differences between DS and OS were related to individual locations of the active electrode contacts. We hypothesized that the focused stimulation of DS could achieve similar motor symptom efficacy while reducing non-motor side effects.

## Results

Recruitment began in 2018, and the final follow-up was completed in 2020. Nineteen (6 female) of 23 enrolled patients completed the study, at which point the trial ended. The remaining 4 dropped out of the trial voluntarily. Those who completed the study had a mean (±standard deviation) age of 63.60 (±8.28) years and disease duration of 11.00 (±3.88) years.

**Fig. 1:**
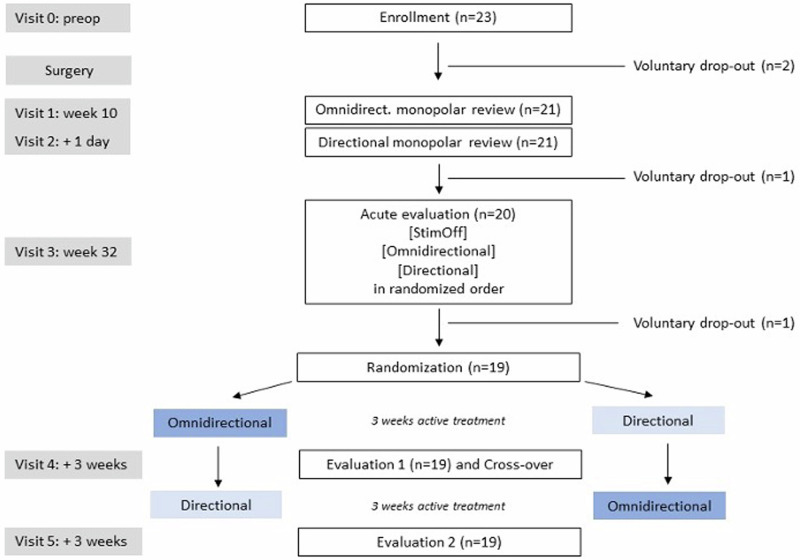
Consort flow diagram of the SANTOP study. A graphical depiction of the study design with the number of patients at each stage.

At 6 months postoperatively (Fig. 1), optimal ring and segmented contacts were compared to each other with mean TEED-adapted stimulation intensities of 2.59 ± 0.77 mA for OS and 1.82 ± 0.61 mA for DS, a difference of 30.37 ± 8.47% (Table 1). First, OS and DS were compared to each other and to Stim OFF in a randomized, cross-over order in an acute setting (V3), i.e., 30 min after reprogramming within the same day. After this acute assessment, OS and DS were compared to each other in a randomized, cross-over order in a chronic/long-term setting, i.e., 3 weeks after reprogramming for each condition (V4/5). Note that while we attempted to match TEED between conditions, three patients required increased stimulation amplitude in the DS condition (one between V3 and V4, two between V4 and V5). There were no significant differences between OS and DS in the primary (objectively quantified muscle rigidity) and secondary (clinical motor and non-motor assessments) outcome parameters.

**Table 1:** Patient demographics and stimulation parameters are summarized

ID	Sex/gender	Age (years)	Disease duration (years)	LEED (mg/d)	Stimulation parameters Omnidirectional	Stimulation parameters Directional
1	m	65	10	1125	L 3− G+ 2.1 mA 60 µs 130 Hz R 11− G+ 3.7 mA 60 µs 130 Hz	L 3C− G+ 1.4 mA 60 µs 130 Hz R 11B− G+ 2.3 mA 60 µs 130 Hz
2*	f	61	15	869	L 3− G+ 3.3 mA 60 µs 130 Hz R 11− G+ 3.5 mA 60 µs 130 Hz	L 3A− G+ 2.5 mA 60 µs 130 Hz R 11C− G+ 2.5 mA 60 µs 130 Hz
3	m	58	11	1250	L 3− G+ 1.3 mA 60 µs 130 Hz R 11− G+ 2.3 mA 60 µs 130 Hz	L 3C− G+ 0.5 mA 60 µs 130 Hz R 11A− G+ 1.2 mA 60 µs 130 Hz
4*	m	71	15	450	L 3− G+ 2.70 mA 60 µs 130 Hz R 11− G+ 2.20 mA 60 µs 130 Hz	L 3A− G+ 2.2 mA 60 µs 130 Hz R 11A− G+ 2.0 mA 60 µs 130 Hz
5	m	58	9	1205	L 3− G+ 1.5 mA 60 µs 130 Hz R 11− G+ 1.5 mA 60 µs 130 Hz	L 3A− G+ 0.95 mA 60 µs 130 Hz R 11C− G+ 1.05 mA 60 µs 130 Hz
6	m	56	6	1293	L 3− G+ 0.8 mA 60 µs 130 Hz R 11− G+ 4.5 mA 60 µs 130 Hz	L 3A− G+ 0.55 mA 60 µs 130Hz R 11A− G+ 3.1 mA 60 µs 130 Hz
7	m	46	11	1265	L 3− G+ 1.6 mA 60 µs 130 Hz R 11− G+ 3.7 mA 60 µs 130 Hz	L 3A− G+ 1.25 mA 60 µs 130 Hz R 11B− G+ 2.85 mA 60 µs 130 Hz
8*	f	68	12	815	L 3− G+ 2.2 mA 60 µs 130 Hz R 11− G+ 4.0 mA 60 µs 130 Hz	L 3A− G+ 1.8 mA 60 µs 130Hz R 11C− G+ 2.7 mA 60 µs 130 Hz
9	f	53	14	375	L 3− G+ 2.8 mA 60 µs 130 Hz R 11− G+ 2.9 mA 60 µs 130 Hz	L 3A− G+ 2.05 mA 60 µs 130 Hz R 11B− G+ 1.95 mA 60 µs 130 Hz
10	f	75	13	725	L 3− G+ 4.7 mA 60 µs 130 Hz R 11− G+ 4.7 mA 60 µs 130 Hz	L 3B− G+ 3.35 mA 60 µs 130Hz R 11B− G+ 3.35 mA 60 µs 130 Hz
11	f	59	18	525	L 3− G+ 1.1 mA 60 µs 130 Hz R 11− G+ 1.5 mA 60 µs 130 Hz	L 3B− G+ 0.75 mA 60 µs 130Hz R 11C− G+ 1.00 mA 60 µs 130 Hz
12	m	61	6	441	L 3− G+ 2.7 mA 60 µs 130 Hz R 11− G+ 2.8 mA 60 µs 130 Hz	L 3C− G+ 2.6 mA 60 µs 130 Hz R 11A− G+ 1.95 mA 60 µs 130 Hz
13	m	73	10	1033	L 3− G+ 2.5 mA 60 µs 130 Hz R 11− G+ 2.7 mA 60 µs 130 Hz	L 3B− G+ 1.7 mA 60 µs 130 Hz R 11B− G+ 1.85 mA 60 µs 130 Hz
14	m	71	8	875	L 3− G+ 3.0 mA 60 µs 130 Hz R 11− G+ 2.8 mA 60 µs 130 Hz	L 3C− G+ 2.1 mA 60 µs 130 Hz R 11A− G+ 1.8 mA 60 µs 130 Hz
15	m	67	12	815	L 2− G+ 1.7 mA 60 µs 130 Hz R 10− G+ 2.2 mA 60 µs 130 Hz	L 2C− G+ 1.1 mA 60 µs 130 Hz R 10B− G+ 1.4 mA 60 µs 130 Hz
16	m	67	18	960	L 3− G+ 2.8 mA 60 µs 130 Hz R 11- G + 3.3 mA 60 µs 130 Hz	L 3B− G+ 2.1 mA 60 µs 130 Hz R 11B− G+ 2.3 mA 60 µs 130 Hz
17	f	52	8	708	L 3− G+ 2.3 mA 60 µs 130 Hz R 11− G+ 2.1 mA 60 µs 130 Hz	L 3C− G+ 1.65 mA 60 µs 130Hz R 11C− G+ 1.5 mA 60 µs 130 Hz
18	m	50	9	859	L 3− G+ 2.7 mA 60 µs 130 Hz R 11− G+ 0.9 mA 60 µs 130 Hz	L 3A− G+ 1.9 mA 60 µs 130Hz R 11B− G+ 0.5 mA 60 µs 130 Hz
19	m	67	13	1050	L 3− G+ 2.7 mA 60 µs 130 Hz R 11− G+ 2.5 mA 60 µs 130 Hz	L 3C− G+ 1.8 mA 60 µs 130 Hz R 11C− G+ 1.7 mA 60 µs 130 Hz

*LEED* levodopa equivalent dose during the cross-over phase.

The asterisk “*” indicates patients in whom a reprogramming was necessary during the cross-over phase: ID2: the calculated directional stimulation was L 3A− G+ 2.1 mA 60 µs 130 Hz, R 11C− G+ 2.2 mA 60 µs 130 Hz; patient reported bradykinesia and had rigidity and freezing of gait with directional stimulation during the cross-over-phase so that we needed to increase the stimulation Intensity ID4: the calculated directional stimulation was L 3A− G+ 1.85 mA 60 µs 130 Hz, R 11A− G+ 1.55 mA 60 µs 130 Hz; patient reported bradykinesia and had rigidity with directional stimulation during the cross-over-phase so that we needed to increase the stimulation intensity. ID8: the calculated directional stimulation was L 3A− G+ 1.6 mA 60 µs 130 Hz, R 11C− G+ 2.6 mA 60 µs 130 Hz; patient reported worsening of motor fluctuations so that we needed to increase the stimulation intensity.

### Motor symptoms

In the *acute setting* (V3), both conditions had similar therapeutic efficacy on motor symptoms, i.e., there was no significant difference between OS and DS (Table 2). Specifically, OS (30.00 ± 13.55; *p* = 0.001) and DS (30.72 ± 10.87; *p* = 0.003) improved UPDRS III as compared to Stim OFF (39.63 ± 12.92); also, the FoG-AC improved with both OS (14.83 ± 13.66; *p* = 0.021) and DS (13.92 ± 13.32; *p* = 0.024) as compared to Stim OFF (17.83 ± 15.05). However, there were no significant differences between OS and DS for UPDRS III (*p* = 0.521) and FoG-AC (*p* = 0.591), or the other outcome measures including the oddball and working memory tasks (VDR/SDR).

**Table 2:** Six months after surgery (V3), both conditions (OS and DS) showed significant therapeutic efficacy with regard to segmental (MDS-UPDRS III) and axial motor (FOG-AC) symptoms compared to Stim Off during the acute evaluation (i.e., 30 min stimulation periods); however, there were no significant differences between the omnidirectional (OS) and directional (DS) conditions

	Omnidirectional	Directional	Stim Off	Stim Off vs. Omnidirectional			Stim Off vs. Directional			Omnidirectional vs. Directional		
	Mean ± SD	Mean ± SD	Mean ± SD	*p* value	*z* value	*n*	*p* value	*z* value	*n*	*p* value	*z* value	*n*
MDS-UPDRS III	30.00 ± 13.55	30.72 ± 10.87	39.63 ± 12.92	**0.001**	−3.298	15	**0.003**	−2.985	15	0.521	−0.641	17
VDR												
Task accuracy	0.71 ± 0.27	0.69 ± 0.27	0.65 ± 0.31	0.181	−1.338	15	0.440	−0.772	16	0.860	−0.176	16
Task reaction time	2.56 ± 0.63	2.51 ± 0.59	2.56 ± 0.38	0.379	−0.879	14	0.532	−0.625	15	0.925	−0.094	15
Oddball accuracy	0.94 ± 0.07	0.90 ± 0.21	0.89 ± 0.17	0.676	−0.418	15	1.000	0.000	16	0.324	−0.986	16
Oddball reaction time	0.92 ± 0.23	0.90 ± 0.26	1.18 ± 0.51	0.061	−1.875	15	**0.002**	−3.131	16	0.227	−1.087	16
SDR												
Task accuracy	0.27 ± 0.10	0.30 ± 0.07	0.29 ± 0.08	0.553	−0.593	15	0.288	−1.063	14	0.475	−0.714	15
Task reaction time	1.76 ± 0.78	1.73 ± 0.60	1.80 ± 0.68	1.000	0.000	15	0.925	−0.094	14	0.532	−0.625	15
Oddball accuracy	0.64 ± 0.22	0.64 ± 0.21	0.65 ± 0.23	0.864	−0.171	15	0.861	−0.175	14	0.527	−0.633	15
Oddball reaction time	1.04 ± 0.41	1.04 ± 0.34	1.11 ± 0.42	0.826	−0.220	14	0.363	−0.910	14	0.875	−0.157	14
FoG-AC	14.83 ± 13.66	13.92 ± 13.32	17.83 ± 15.05	**0.021**	−2.307	12	**0.024**	−2.251	12	0.591	−0538	12
CAPSIT												
Steps	38.36 ± 22.26	43.50 ± 33.56	38.75 ± 26.29	0.119	−1.561	12	0.285	−1.069	12	0.166	−1.385	14
Time (s)	33.07 ± 43.95	37.86 ± 58.97	37.58 ± 51.46	0.073	−1.795	12	0.349	−0.936	12	0.244	−1.166	14
Freezing episodes	0.93 ± 1.73	2.50 ± 5.97	1.67 ± 3.42	0.273	−1.095	12	1.000	0.000	12	0.225	−1.214	14

Significant *p* values (<0.05) are in bold.

In the long-term setting (V4/5), both conditions also had similar therapeutic efficacy, i.e., there were no significant differences between OS and DS with regard to motor symptoms (Table 3) and EMG-based assessments (*p* ≥ 0.135). However, in 3 of the 19 patients (i.e., 15.79%), stimulation amplitudes had to be increased in the DS condition (above and beyond the estimated TEED) to achieve the therapeutic current strength, i.e., to avoid side effects such as motor fluctuations, bradykinesia, rigidity, and freezing of gait (see Table 1 legend for further details).

**Table 3:** Six months after surgery, in the cross-over period (V4/5), there were no significant differences between OS and DS with regard to motor and non-motor symptoms during the chronic evaluation (i.e., after 3-week stimulation blocks of each condition)

	Omnidirectional	Directional	Omnidirectional vs. Directional		
	Mean ± SD	Mean ± SD	*p* value	*z* value	*n*
MDS-UPDRS I	11.16 ± 8.49	11.00 ± 5.94	0.909	−0.114	17
MDS-UPDRS II	18.16 ± 9.32	19.06 ± 9.19	0.568	−0.571	18
MDS-UPDRS III	28.63 ± 12.91	32.42 ± 15.07	0.121	−1.550	19
MDS-UPDRS IV	8.17 ± 4.89	8.78 ± 4.45	0.925	−0.095	17
VDR					
Task accuracy	0.70 ± 0.29	0.69 ± 0.23	1.000	0.000	17
Task reaction time	2.56 ± 0.51	2.54 ± 0.59	0.845	−0.196	18
Oddball accuracy	0.96 ± 0.08	0.94 ± 0.11	0.271	−1.100	18
Oddball reaction time	0.91 ± 0.32	0.85 ± 0.27	0.267	−1.111	18
SDR					
Task accuracy	0.25 ± 0.12	0.22 ± 0.09	0.355	−0.924	17
Task reaction time	1.83 ± 0.64	1.71 ± 0.53	0.918	−0.103	16
Oddball accuracy	0.64 ± 0.26	0.57 ± 0.26	0.265	−1.115	17
Oddball reaction time	1.09 ± 0.35	1.01 ± 0.33	0.469	−0.724	16
FoG-AC	11.47 ± 13.02	12.80 ± 13.80	0.129	−1.517	15
CAPSIT					
Steps	39.11 ± 20.55	42.00 ± 22.02	0.072	−1.801	17
Time (s)	27.44 ± 23.74	31.82 ± 34.04	0.142	−1.468	17
Freezing episodes	1.56 ± 3.75	1.53 ± 3.63	0.131	−1.511	17
MoCA	26.53 ± 3.15	27.83 ± 1.92	0.080	−1.753	17
Executive	3.89 ± 1.05	4.47 ± 0.84	0.071	−1.805	19
Naming	2.84 ± 0.69	3.00 ± 0.00	0.317	−1.000	18
Attention	5.58 ± 0.69	5.44 ± 0.86	0.206	−1.265	18
Language	2.44 ± 0.62	2.32 ± 0.82	0.366	−0.905	18
Abstraction	1.89 ± 0.32	2.00 ± 0.00	0.157	−1.414	18
Delayed recall	3.95 ± 1.31	4.37 ± 0.83	0.323	−0.988	19
Orientation	5.95 ± 0.23	6.00 ± 0.00	0.317	−1.000	19
BDI	13.56 ± 7.25	14.88 ± 6.91	0.448	−0.758	17
AS	16.11 ± 4.58	17.58 ± 4.49	0.646	−0.406	16
DBS-IS	25.94 ± 14.61	28.06 ± 12.52	0.674	−0.421	17
LARS	−22.47 ± 7.33	−20.61 ± 10.14	0.275	−1.091	18
PDQ-39					
Mobility	19.22 ± 10.73	21.13 ± 8.16	0.380	−0.877	15
Activities of daily living	9.06 ± 5.68	9.72 ± 5.06	1.000	0.000	17
Emotions	8.06 ± 4.56	9.00 ± 4.04	0.318	−0.999	17
Stigma	3.50 ± 3.24	4.39 ± 2.43	0.304	−1.027	17
Social support	1.94 ± 2.31	2.53 ± 2.27	0.094	−1.672	17
Cognition	5.00 ± 3.27	6.38 ± 2.90	0.477	−0.711	15
Communication	4.06 ± 3.42	4.53 ± 2.74	0.832	−0.212	16
Bodily discomfort	5.11 ± 2.61	5.35 ± 2.34	0.640	−0.467	16

**Fig. 2:**
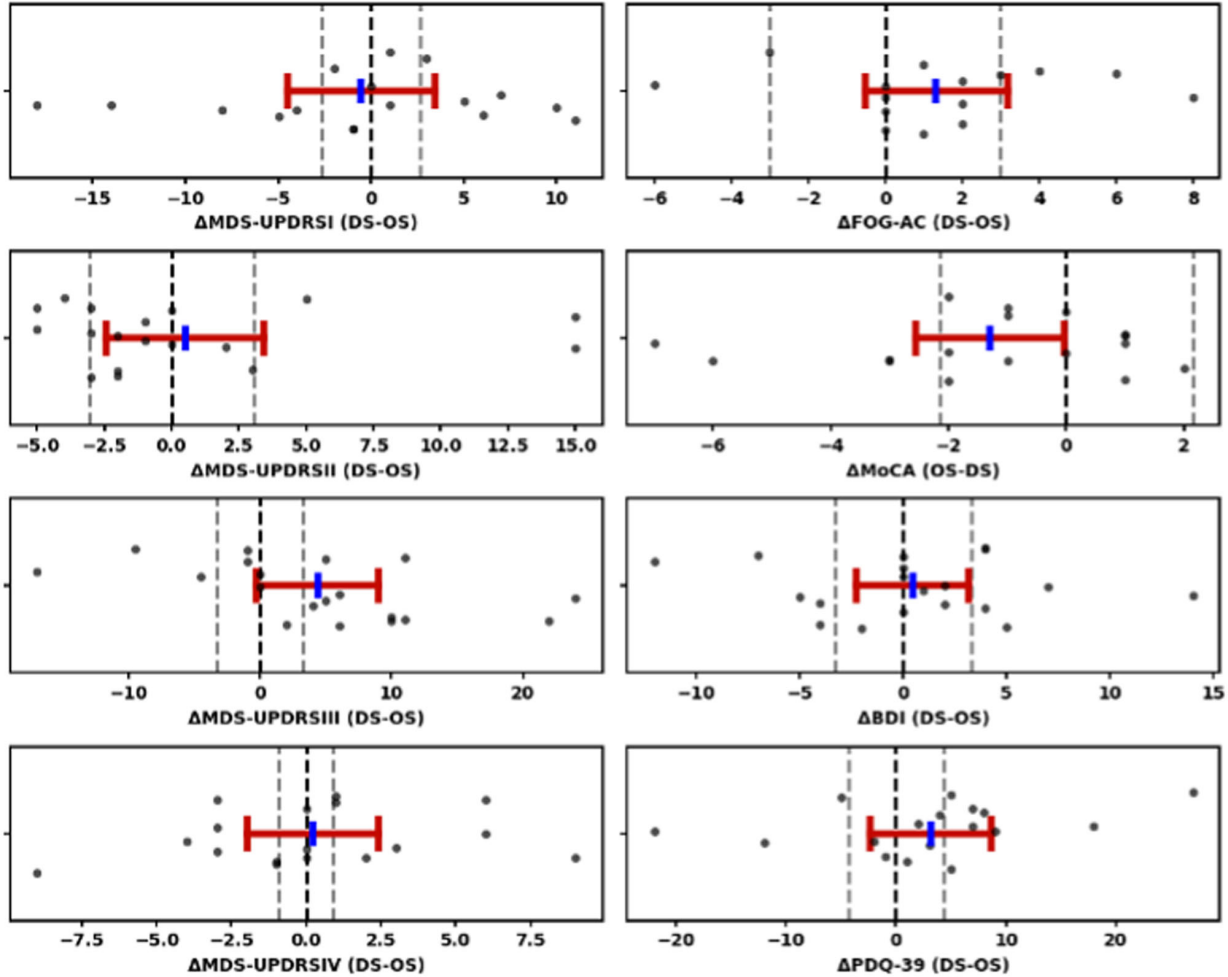
Ninety-five percent confidence interval plots for secondary outcomes. The 95% confidence intervals (CI) for the mean difference are plotted for motor and non-motor scores (V4/5 data). Differences for individual patients are shown with black dots. A CI that lies entirely to the left or right of the zero-line (black) indicates that DS is superior to OS, or OS is superior to DS, respectively. The gray dashed lines represent margins based on minimal clinically important differences (MCIDs). A CI that lies entirely on the left side of the right gray line indicates that DS is non-inferior to OS. A CI that lies entirely on the right side of the left gray line indicates that OS is non-inferior to DS. A CI that lies entirely between the two gray lines indicates equivalence between DS and OS. Please note that in this figure, MoCA is the only score where performance improves with higher values. Consequently, the delta calculation had to be reversed, i.e. OS–DS instead of DS–OS, in order to maintain consistency between scores.

Notably, OS scored (non-significantly) better than DS for UPDRS III (28.63 ± 12.91 vs. 32.42 ± 15.07), FoG (11.47 ± 13.02 vs. 12.80 ± 13.80), CAPSIT steps (39.11 ± 20.55 vs. 42.00 ± 22.02), CAPSIT time (27.44 ± 23.74 vs. 31.82 ± 34.04), and PDQ-39 Mobility (19.22 ± 10.73 vs. 21.13 ± 8.16). In addition, the long-term stimulation (i.e., evaluation after 3-week blocks, V4/5) changed the scores compared to the acute evaluation (i.e., evaluation after 30 min, V3); specifically, the UPDRS III score improved from 30.00 ± 13.55 to 28.63 ± 12.91 for OS, and deteriorated from 30.72 ± 10.87 to 32.42 ± 15.07 for DS. This effect in favor of OS was supported when comparing the 95% confidence interval (CI) around the mean difference for FOG and UPDRS III with the minimal clinically important difference (MCID) thresholds and the zero-lines (Fig. 2).

### Non-motor symptoms

In the long-term setting (V4/5), there were no significant differences between OS and DS with regard to non-motor symptoms (Table 3). Notably, OS scored (non-significantly) better than DS on all (BDI, AS, DBS-IS, LARS, PDQ-39) but one non-motor clinical measure. The only non-motor clinical measure that favored DS was the MoCA score; specifically, OS resulted in (non-significantly) more cognitive impairment, i.e., a lower MoCA score as compared to DS (26.53 ± 3.15 vs. 27.83 ± 1.92; *p* = 0.080). This effect was driven by a deterioration of the MoCA subscore for executive functions (OS vs. DS: 3.89 ± 1.05 vs. 4.47 ± 0.84; p = 0.071; OS vs. preoperative baseline: 3.89 ± 1.05 vs. 4.63 ± 0.60; *p* = 0.015). This effect in favor of DS was supported when comparing the 95% CI around the mean difference for MoCA with the MCID threshold and the zero-line (Fig. 2).

Since MoCA was administered twice, both at V4 and V5, we tested for practice effects. A one-way ANOVA comparing total MoCA between V4 and V5 with subject as a blocking variable showed a significant effect of test session (*F* = 10.51, *p* = 0.0045).

### Executive function

**Fig. 3:**
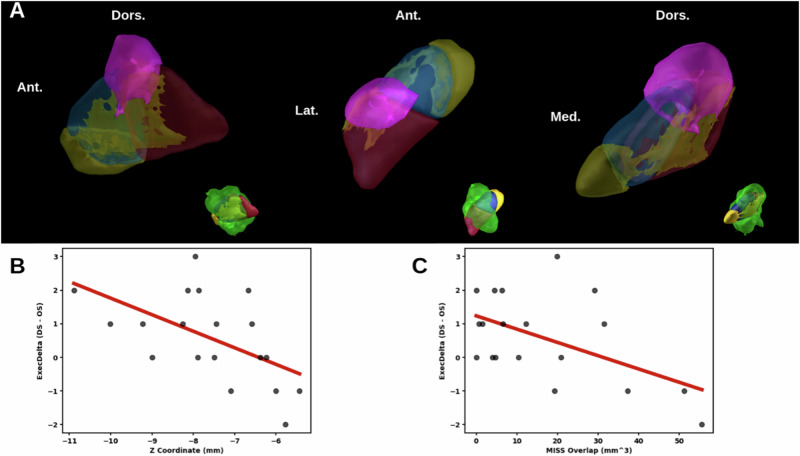
Executive benefits of DS over OS depend on electrode location and MISS engagement. **A** A thresholded t-image based on ExecDeltas and OS VTAs is shown in magenta. This region indicates a volume where DS offers less or no benefit over OS. The motor, associative, and limbic regions of the STN are shown in red, blue, and yellow respectively. The insets show in green all voxels that were stimulated by at least one VTA. **B** The correlation between the MNI Z coordinate of the active contact and the ExecDelta is shown. **C** The correlation between omnidirectional stimulation VTA–MISS overlap and ExecDelta is shown.

To better understand the potential differences between OS and DS on executive function, we sought to identify factors that influence when DS is beneficial and when it is not. Each patient’s “ExecDelta” is their executive function on DS minus OS. When ExecDelta was mapped onto modeled volumes of tissue activated (VTAs) of OS, we identified a region where stimulation was associated with less or no improvement with DS compared to OS (Fig. 3A). This region was located at the dorsal border of the STN^29^. Indeed, we found that patients with more dorsally active contacts derived less benefit from DS in terms of their executive function compared to OS (*r*^2^ = 0.30, *F* = 7.4, *p* = 0.0145; Fig. 3B). Furthermore, this region overlapped with the motor improvement sweet spot (MISS; 54). The less the VTA of OS overlapped with the MISS, the more patients benefited from DS relative to OS (*r*^2^ = 0.27, *F* = 6.3, *p* = 0.0225; Fig. 3C).

We repeated all our VTA analyses, this time using DS VTAs instead of OS (Fig. S1A). The outcome of the DS analysis was highly similar to that of OS (overlap with MISS predicts lower ExecDelta; *r*^2^ = 0.29, *F* = 7.0, *p* = 0.0168), which is unsurprising, given that DS and OS VTAs are correlated in space, having largely overlapping VTAs. In addition, we show that the areas spared by DS in the ten patients who improve with this treatment are located at the ventro-medial and ventro-lateral boundary of the STN (Fig. S1B).

## Discussion

The objective of this prospective, randomized clinical trial was to determine whether directional steering with segmented contacts could improve the accuracy of subthalamic DBS by reducing stimulation intensity for motor symptom relief and avoiding nearby areas responsible for side effects.

As hypothesized, there were no significant differences between OS and DS with regard to primary (EMG assessment of muscle rigidity) and secondary (clinical motor assessment) outcome parameters for motor symptom relief. Both OS and DS significantly improved motor scores compared to no stimulation, with DS requiring 30% less stimulation intensity. After chronic stimulation at V4/5, motor performance increased under OS relative to V3 measurements, whereas it worsened under DS. This resulted in a clinically important mean difference between OS and DS during the chronic assessment, which sometimes required increasing DS intensity beyond the adjusted estimates of the TEED to achieve OS benefits. Notably, OS scored (non-significantly) better than DS in all clinical motor assessments. While there might be a carry-over effect influencing acute V3 measurements (due to 6-month OS from V2 to V3), there is no reason to suspect that these would preferentially impact no stimulation, OS, or DS conditions.

There were also no significant differences between OS and DS with regard to the secondary outcome parameters for non-motor symptoms such as cognition, mood, and quality of life. Notably, OS scored (non-significantly) better than DS on all but one non-motor clinical measure. Specifically, OS resulted in (non-significantly) more cognitive impairment than DS (*p* = 0.080), an effect that was driven by a deterioration of executive functions (OS vs. DS: *p* = 0.071; OS vs. preoperative baseline: *p* = 0.015), which is known to be particularly affected in PD^30^ and by subthalamic DBS^31^. Exploratory analyses showed that the more ventral the active electrode contact and the less the volume of activated tissue overlapped with the motor improvement sweet spot, the greater the benefit of DS over OS for executive function.

Currently, there are few systematic studies comparing directional DBS with standard omnidirectional stimulation, including intra- and extra-operative assessments. One intraoperative study^14^ showed a larger therapeutic window with DS, increasing the distance between the clinical efficacy threshold and the side effect threshold. However, another intraoperative study^13^ found no relevant increase in therapeutic window with DS. Two extra-operative studies (a retrospective, open-label pilot study^16^ and a prospective, randomized, double-blind study^15^) reported an increased side effects threshold but no decrease in the clinical efficacy threshold. Another retrospective, open-label case series showed a decrease in the clinical efficacy threshold with DS^32^. This latter study used different step sizes for OS (0.5 mA) and DS (0.1–0.2 mA) to account for the higher charge density of DS. This may have biased the sensitivity to detect the clinical efficacy threshold in favor of DS. Therefore, accurate thresholding with the same amplitude step sizes for both conditions is critical. Furthermore, differences between segmented stimulation directions have been found in side effect thresholds^16^, but not in therapeutic efficacy^14^. These variable results may be due to different accuracy of stimulation amplitude titration and subjective symptom rating on an ordinal scale (UPDRS III).

The most comprehensive study to date is the prospective, randomized, double-blind, multicenter PROGRESS trial^17^. It showed that DS resulted in a wider therapeutic window compared to OS 3 months after initial programming. In addition, DS reduced the current required for symptom relief compared to OS^17^. However, like previous studies, it lacked a systematic assessment of non-motor symptoms and relied on subjective clinical assessments using the UPDRS ordinal scale. Stimulation titration protocols varied between centers and TEED was not maintained over time. The 3-month open-label observations had a fixed order, with OS always preceding DS.

The present study sought to address the limitations of previous directional DBS studies by comparing OS and DS not only with subjective clinical assessment but also with objective EMG assessment of muscle rigidity in a randomized, double-blind fashion. In addition, stimulation parameters were titrated in small increments (0.2 mA) for both OS and DS, and TEED was maintained for 3 weeks for each condition.

This approach demonstrated a significant improvement in the UPDRS III score with OS and DS in the acute setting. In the chronic setting, the score continued to improve with OS but worsened with DS. The mean difference between OS and DS exceeded the 3.25-point threshold for a MCID of the UPDRS III^33^. Similarly, FoG-AC demonstrated significant improvement with both OS and DS in the acute assessment, with continued improvement observed in the chronic setting. When applying OS, the mean difference between the acute and chronic settings exceeded the three-point threshold for a MCID of the FoG-AC^34^. These findings with regard to the MCIDs, although not statistically significant due to the high variability of the changes, indicate that OS may provide more sustained motor benefits over time. They also highlight the need for clinical evaluations beyond acute assessments to reliably capture differences between OS and DS. In addition, the variability in patient responses calls for further investigation with larger sample sizes to confirm the observed trends.

Furthermore, in 3 of the 19 patients, DS required increased electrical energy above the estimated TEED during the chronic/long-term period to achieve the therapeutic motor benefits of OS. These findings also suggest that longer evaluation periods are needed to capture the full benefit and potential downsides of different programming approaches. These findings may be related to the different VTA by OS vs. DS. Computational modeling suggested that TEED-balanced DS, with ~30% less stimulation amplitude than OS, reduced total VTA by 15%^28^. In the present study, DS likely resulted in reduced coverage of targeted and non-targeted volumes in the STN, resulting in fewer motor benefits and fewer executive function side effects, respectively.

In light of these considerations, we examined changes in executive function in relation to the VTA, assuming that patients with less optimally located active contacts would benefit more from DS. Compared to baseline, patients with OS but not DS experienced a significant decline in executive function. This decline was reversed by DS the more ventral the active contact was located (Fig. 3B) and the less the VTA overlapped with the previously described^35^ motor improvement sweet spot (Fig. 3C). These findings complement previous approaches that reduced working memory decline (quantified by an n-back task) by using patient-specific models to optimize stimulation of the dorsal STN and adjacent white matter, while minimizing stimulation of non-motor regions^36^. In another study, a normative connectome was applied to the same patient group and showed that the estimated functional connectivity circuit of each patient’s stimulation volume was more predictive of cognitive dysfunction in an independent cohort than the stimulation volume itself^37^. The present study may offer actionable insights for creating effective clinical strategies to maximize patient benefit in daily practice, even without sophisticated imaging and modeling. It suggests that patient-reported measures such as the MoCA questionnaire acquired before and after a 3-week stimulation period may be sufficient to identify DBS-induced executive dysfunction. Furthermore, it indicates that choosing a more dorsal electrode contact and/or directional steering might be sufficient to reverse this effect.

Despite our efforts to account for numerous factors in order to facilitate a systematic comparison of DS and OS, this study is not without limitations. The study was powered for the primary outcome measure, i.e., objectively quantified muscle rigidity, but not for the secondary outcome measures of motor and non-motor assessments. Therefore, no conclusions can be drawn regarding clinically meaningful differences in the reported observations.

The sample size, while comparable to that of other single-center studies, is smaller than that of the multicenter PROGRESS study. This may have prevented the numerical differences that were present between OS and DS after the 3-week evaluation periods in both motor and non-motor measures from reaching statistical significance in favor of OS. Notably, the general trend favoring OS was contradicted by findings on executive functions, which favored DS. Although this result may be theoretically influenced by multiple comparisons, the dependence of this effect on the overlap with the motor improvement sweet spot indicates that the differential impact of OS and DS on executive function is likely genuine and not merely a statistical artifact. This promising finding should be validated by confirmatory studies with larger sample sizes. In this context, the findings of the present study may be informative for future sample size estimations.

A notable advantage of this approach is that each patient underwent consistent and detailed stimulation programming and parameter titration using small current intensity increments. However, this titration was based on the standard clinical procedure, which focused on one symptom, specifically rigidity. This may have prevented us from identifying the optimal programming for other symptoms. Furthermore, although EMG provided objective measures for the symptom targeted during programming, variability in EMG electrode placement may have obscured differences between conditions. Nevertheless, the results were consistent with standard subjective ordinal scale-based assessments, such as UPDRS III scores.

A distinctive feature of this study is that TEED was sustained across both stimulation conditions for an extended period. This was achieved by DS utilizing a lower current intensity than OS, which was below the threshold for side effects. This approach permitted an unbiased comparison with respect to the electrical energy, and thus it did not allow for a direct comparison of each condition at its maximum current intensity. The results indicate that, when used within the lower current limits, DS can attain comparable efficacy to OS, despite OS demonstrating a numerically greater effect. It remains unclear whether DS at maximum intensity may have provided superior benefits. Future studies may consider comparing OS and DS at the same stimulation intensities, despite the greater energy burden of the latter for battery lifespan, since the availability of rechargeable impulse generators minimizes the frequency of subsequent surgical procedures for battery replacement. In addition, the 3-week observation period for each condition may not have been sufficient to detect more pronounced differences, so that, for example, 3-month periods may be considered in the future.

Practice effects may have influenced MoCA scores between V4 and V5. However, because the order of OS and DS was balanced across patients, it is improbable that learning effects were a factor in the differences we observed between treatment conditions.

Since cognitive effects of DBS tend to have a longer time course than motor effects, MoCA scores may reflect, in part, a carryover from 6 months of OS prior to V3. Therefore, comparing V4/5 OS and DS scores directly to pre-stimulation baseline should be done with that caveat in mind. However, because the initial 6 months of OS was common to both treatment arms, and because the order of OS/DS was balanced across patients, differences between OS and DS at V4/5 are likely not being driven by carry-over effects.

In conclusion, this study indicated no significant differences between OS and DS with respect to primary (muscle rigidity EMG) and secondary (clinical assessment) outcomes for motor symptom relief, while DS required 30% less stimulation intensity. However, the motor improvements observed with DS showed a tendency to deteriorate over time, at times necessitating a higher intensity than the adjusted estimates of the TEED to match the benefits observed with OS. Overall, OS demonstrated (non-significant) superior outcomes compared to DS across all clinical motor and non-motor assessments. However, there was a notable exception in the increased cognitive impairment with OS, particularly in executive functions, as compared to baseline exploratory analyses indicated that DS may ameliorate this executive dysfunction when active electrode contacts are more ventral and the volume of tissue activated with OS is less overlapped with the motor improvement sweet spot.

## Methods

### Patients

All patients who participated in this study were nominated for DBS surgery upon multidisciplinary review based on standard inclusion/exclusion criteria for STN DBS in PD^38^, independent from study participation. Ages eligible for the study were 18–80 years. Exclusion criteria were pregnancy, cognitive impairment (Mini Mental State Exam < 20), suicidality, psychosis, or other severe pathological chronic conditions that might confound treatment effects or interpretation of the data. This study was conducted in accordance with the Declaration of Helsinki and approved by the ethics committee of the Medical Faculty of the University of Tübingen (700/2017B01). Twenty-three consecutive akinetic-rigid patients scheduled for STN DBS were included in this study comparing DS with OS after providing written informed consent to participation.

The primary outcome measure was objectively quantified muscle rigidity of the upper extremity, i.e., surface EMG recordings of the biceps and triceps muscles during standardized extension/flexion of the elbow joint^39^ assessed 6 months after implantation in a cross-over design after stimulation titration in small increments (0.2 mA steps) for both OS and DS. The study was designed to have 80% power to detect a 0.27 mA change in therapeutic stimulation threshold with a two-tailed *P* < 0.05 (Wilcoxon rank sum test).

Secondary outcome measures included a large battery of clinical motor and non-motor symptoms in an exploratory approach (Table 3). The study was not powered to detect significant differences between OS and DS on these secondary outcome measures after correction for multiple comparisons. The intention to investigate these additional outcome parameters was exploratory, to identify trends and clinical areas for image-based ancillary analyses (i.e., location of active contacts to reveal stimulation-behavior relationships) to inform subsequent confirmatory studies. However, the calculated sample size of 20 patients for the primary outcome parameter may still have been sufficient to detect significant differences in some, but not all, secondary outcome parameters based on previous work^14^. All data were collected at the Tübingen University Hospital.

Four patients dropped out due to personal reasons not related to the study. Nineteen patients completed the study with the cross-over treatment evaluation periods. There were no changes to methods or outcome criteria after the trial commenced. Participant and drop-out numbers at each phase of the study are summarized in Fig. 1. Patient demographics are summarized in Table 1.

### Surgical procedure

The tentative implantation location in each hemisphere was initially determined by conventional image-based direct targeting of the STN using preoperative MRI (T2-weighted and/or SWI)^40^. Patients were withdrawn from antiparkinsonian medications overnight, and on the day of surgery electrophysiological recordings of single-unit and/or local field potential activities were used to confirm the target location in each hemisphere. Initially, single-unit recordings were used to delineate STN entry and spatial extent by characteristic burst firing rates (20–60 Hz) and irregular or bursting patterns^41^. The trajectory yielding greatest spatial extent of STN was then selected for the DBS electrode implant. The final position of the DBS electrode was once again titrated using neurophysiological recordings directly from the DBS contacts, such that two levels of the DBS lead-containing segmented contacts were placed in the area of STN yielding greatest beta (13–30 Hz) oscillatory power, determined by (online) intraoperative power spectral density calculations^21^. There were no complications from surgical procedures.

### Study design

This study was a prospective, randomized, double-blind, cross-over, single-center clinical trial, i.e., the Subthalamic steering for therapy optimization in Parkinson’s disease (SANTOP) study (preregistered at ClinicalTrials.gov: NCT03548506 on 2018-06-06). The comprehensive study design is illustrated in Fig. 1. The rationale for the cross-over design was to enable each patient to serve as their own control, thereby reducing the impact of inter-patient variability on the study outcomes, minimizing confounding factors, and facilitating a more nuanced understanding of the relative efficacy and side effect profile of each stimulation mode. The potential for carry-over and period effects in cross-over studies was mitigated through the deliberate selection of a 3-week cross-over period for each stimulation condition. Specifically, the effects of DBS parameter changes are transient, lasting minutes to hours. Therefore, a 3-week period of stimulation in one condition is sufficient to exclude carry-over effects. Furthermore, this time frame is sufficiently brief to preclude the potential influence of disease progression, which must be considered in the context of neurodegenerative disorders such as PD.

The patients were enrolled preoperatively (V0). The postoperative visits started 2 months (V1/2) and 6 months (V3/4/5) after surgery. All evaluations were done in the Med OFF condition after overnight withdrawal of dopaminergic medication. The patients and the evaluators were blinded to stimulation conditions during the whole study. At the 2-month visits, a monopolar review was performed on 2 subsequent days to identify the optimal contact and stimulation parameters for omnidirectional (V1) and directional (V2) stimulation. Stimulation was then set to OS mode between V2 and V3.

At the 6-month visit (V3), a further clinical test was performed to check the therapeutic threshold of stimulation amplitude. This was done to ensure that the settings remained optimal for the patient. TEED was also recalculated at V3, thereby taking into account any possible changes in electrode impedance that may have occurred in the months following the initial monopolar review. This allowed for precise adjustments of the therapy as needed.

Still during V3, OS, and DS were compared to each other and to Stim OFF in randomized order, i.e., 30 min from reprogramming for each condition (acute setting). Afterwards, stimulation settings were randomized again for a 3-week (chronic setting) active treatment period (by a study coordinator who would not be involved in clinical evaluation) to either the omnidirectional settings, or the energy-equivalent directional settings (preconfigured options in the stimulation programmer). After the first 3-week treatment period (V4), blinded collection of outcome data (described below) was performed by a clinician, followed by a cross-over in stimulation settings performed by a study coordinator. After another 3-week treatment period (V5), blinded outcome data were collected once more. This procedure allowed to directly compare OS and DS after long-term stimulation periods, during which other parameters (such as medication) were kept unchanged. These study epochs are summarized in Fig. 1.

### Identification of optimal contact and stimulation parameters

At 2-month (on average 67 days, ±9 days, range 56–88 days) postoperatively (see Fig. 1), stimulation titration was performed on 2 subsequent days after overnight withdrawal from medication. On the 1st day (V1), each patient underwent a monopolar review of the three upper contacts using circular stimulation at each level to determine the optimal OS level. On the 2nd day (V2), the same procedure was repeated using the three-segmented contacts of the previously determined best omnidirectional level (V1) to determine the optimal stimulation direction.

During these visits, rigidity in the contralateral upper limb was assessed (double-blind) by a clinician during unilateral stimulation, while the programming and random electrode contact selection was conducted by another examiner at a stimulation frequency of 130 Hz and a pulse width 60 μs, using intensities starting from 0.5 mA and incrementally increasing by 0.2 mA, until a (self-reported) side effect threshold was reached. At each stimulation increment, a 1-min rigidity assessment (consisting of passive movements of the upper limb by flexing and extending the elbow) was performed to determine the effect threshold.

The therapeutic window was determined for each evaluated contact, and the contact with the greatest therapeutic window and/or best effect threshold in each hemisphere was considered optimal. The stimulation intensity for each optimal segmented contact was then determined by titration of the stimulation intensity such that the TEED^42^ was equivalent to the total electrical energy of the optimal omnidirectional settings. Of note, the stimulation intensity for DS was always lower than for OS due to the greater impedance associated with a smaller stimulation surface (see also the Introduction). The OS and DS parameters for each patient are available in Table 1.

### Treatment evaluation and outcome measures

At a minimum of 6 months (on average 229 days, ±46 days, range 187–369 days) postoperatively (see Fig. 1), OS and DS were compared to each other in an acute (V3) and chronic setting (V4/5). The muscle rigidity of the upper extremity was measured by means of surface EMG recording (BrainAmp DC/EXG, Brain Products, Munich, Germany; sampled at 5 kHz) of the M. biceps brachii and the M. triceps brachii. Previous work suggests that the EMG profile during standardized passive extension and flexion movements of the elbow joint is influenced by DBS and corresponds with standardized clinical scales^39^. The evaluation procedure consisted of EMG recording during a 30-s non-movement period, followed by a 60-s period of passive flexion/extension (~0.5 Hz cycles) of the elbow (~90°). Five trials of this procedure were performed for each arm at each stimulation intensity and each electrode contact.

At both V3 and V4/5, the following data were collected: MDS-UPDRS parts III^43^; timed Capsit-PD walking test^44^; Freezing of Gait Assessment Course (FoG-AC)^45^; Spatial (SDR)^46^ and verbal (VDR)^47^ working memory with respective visual^48^ and acoustic odd-ball tests^49^ for attention monitoring, and task-related EEG recordings^50^. During the acute V3 assessment, no stimulation (STIM OFF), directional, and omnidirectional settings were compared in randomized order. During the chronic V4/5 cross-over assessment, directional and omnidirectional settings were compared in randomized order. The allocation sequence was determined by using a random-number table without blocking. To conceal the sequence until interventions were assigned, generation of the sequence, patient enrollment, and assignment to intervention were conducted by different members of the study team.

At V4/5, this additional data were acquired: MDS-UPDRS part I, II, and IV^43^; Parkinson’s Disease Quality of Life Scale (PDQ-39)^51^; Becks’ Depression Inventory (BDI)^52^; Montreal Cognitive Assessment (MoCA)^53^; Apathy Scale^54^; Lille Apathy rating scale^55^; Deep Brain Stimulation impairment scale (DBS-IS)^56^.

### EMG data processing and analysis

EMG data processing consisted of artifact rejection, epoching, feature extraction, and faulty trial rejection. Initially, the stimulation artifact, the line noise, and their respective harmonics were removed from the data. After artifacts had been removed, the data were split into epochs. From each run, two segments were extracted, a 30-s non-movement segment and a 30-s movement segment. These segments were further divided into individual 2-s epochs. While strict 2-s cutting was applied to the non-movement phase data, the movement phase data were cut according to full movement cycles (0.5 Hz). To extract feature information from the EMG signal, the signal was detrended, and enveloped via the absolute of the Hilbert transform. The average amplitude (power) of the signal was extracted as feature information and was then normalized with respect to the overall EMG power between 2 and 300 Hz to account for different impedance levels across recordings. Finally, outliers on a feature level were rejected if *z* score > 2 (i.e., more than two standard deviations from the mean).

### Volume of tissue activated and changes in executive function

Lead reconstruction in template space and VTA calculation were performed using LeadDBS on all 19 patients who completed the study^57^. All analyses were conducted twice, for OS and DS separately. First, preoperative T1-weighted MRIs were coregistered with postoperative CT scans, followed by subcortical refinement with a coarse mask and transformation to MNI space using advanced normalization tools^58^. The PaCER algorithm^59^ was used to pre-reconstruct DBS electrodes based on lead artifacts in the postoperative CT, followed by manual correction for final localization. In the case of DS VTAs, the directions of segmented contacts were determined using DiODe v2^60^. VTAs were then calculated with a finite element model using SimBio/FieldTrip^61^ with gray and white matter segmentations from the DISTAL Minimal Atlas^29^. Gray and white matter conductivities were set to 0.33 S/m and 0.14 S/m, respectively. The electric field threshold was set at 0.2 V/mm. Correlation of VTAs and active contact coordinates with executive function was performed in LeadGroup^62^. For each patient, we subtracted the MoCA Executive Function Subscore on OS from DS (DS–OS), which we refer to as the ExecDelta. The average *Z* coordinate in MNI space was calculated for each pair of active contacts and correlated (Pearson’s correlation) with the ExecDelta. Additionally, the overlap of each VTA with a previously identified motor improvement sweet spot (MISS; 54) was calculated and correlated (Person’s correlation) with ExecDelta. To visualize regions of activated tissue where OS was more favored relative to DS, each VTA was weighted by its ExecDelta. All VTAs were mirrored to be in the same hemisphere. For each voxel, a *t*-statistic was calculated comparing the ExecDelta for VTAs containing that voxel to the remaining VTAs. The resulting t-image was thresholded at an arbitrary value of 7. All voxels stimulated by at least 1 VTA were included in this visualization (i.e., the *n* threshold was 0).

The algorithm we used to reconstruct segmented contacts for DS (DiODE v2; 65) was validated on a different model of DBS lead from the one used in this study. Furthermore, the algorithm is most accurate when the polar angle of the lead relative to the axis of the CT scanner is <40°; an assumption often not met in our dataset. Due to these limitations, we only emphasize OS results in the main text. Nevertheless, for completeness’ sake, the full results of the DS VTA analysis can be found in a supplement, including those areas that are spared by DS in the patients who improve with this treatment.

### Statistical analyses

All outcome measures were assessed using paired Wilcoxon signed-rank tests to compare stimulation conditions during both acute assessments (Stim OFF vs. OS vs. DS) and chronic assessments (OS vs. DS). Primary EMG outcome data were also analyzed using generalized linear mixed models (glmm). Factors modeled in the analysis were as follows: “counter-movement” to evaluate the effect of the counter-movement on the EMG power; the “stimulation-condition:” to contrast DS and OS modes; “movement” to contrast between rest vs. passive movement phases in EMG activity; the interaction between the stimulation-condition and movement; the (chronologically ordered) “recording id” to compensate for repeated measurements over time; “trial id” to compensate for repeated measurements within trials; “patient id” as a random factor to compensate for repeated measurements across patients. Secondary outcome data of clinical scored were additionally (i.e., outside of the study protocol) compared to preoperative baseline scores, whenever detecting trend-level findings during chronic assessments.

To enhance the clinical interpretability of our findings, we conducted additional analyses on both motor and non-motor scores. For each patient, we calculated the difference between conditions and subsequently determined the 95% CI around the mean difference. These CIs were then compared to both the zero-line and the MCID thresholds as reported in the literature^33,34,54,63,64^ to detect superiority, non-inferiority, and equivalence between DS and OS.

## Supplementary information

Supplemental Material

Consort Checklist

## Data Availability

Data are available upon request.

## Code availability

Code is available upon request.
