# Supplementary material for: Randomized crossover trial on motor and non-motor outcome of directional deep brain stimulation in Parkinson’s disease
Source: NPJ Parkinsons Dis. 2024 Oct 26;10:204. doi: 10.1038/s41531-024-00812-0 (PMC11513109; doi:10.1038/s41531-024-00812-0)
Supplement: Supplementary file 1 — Supplemental Material [file 41531_2024_812_MOESM1_ESM.docx]

**Randomized crossover trial on motor and non-motor outcome of**

**directional deep brain stimulation in Parkinson’s disease**

**Supplemental Figures**


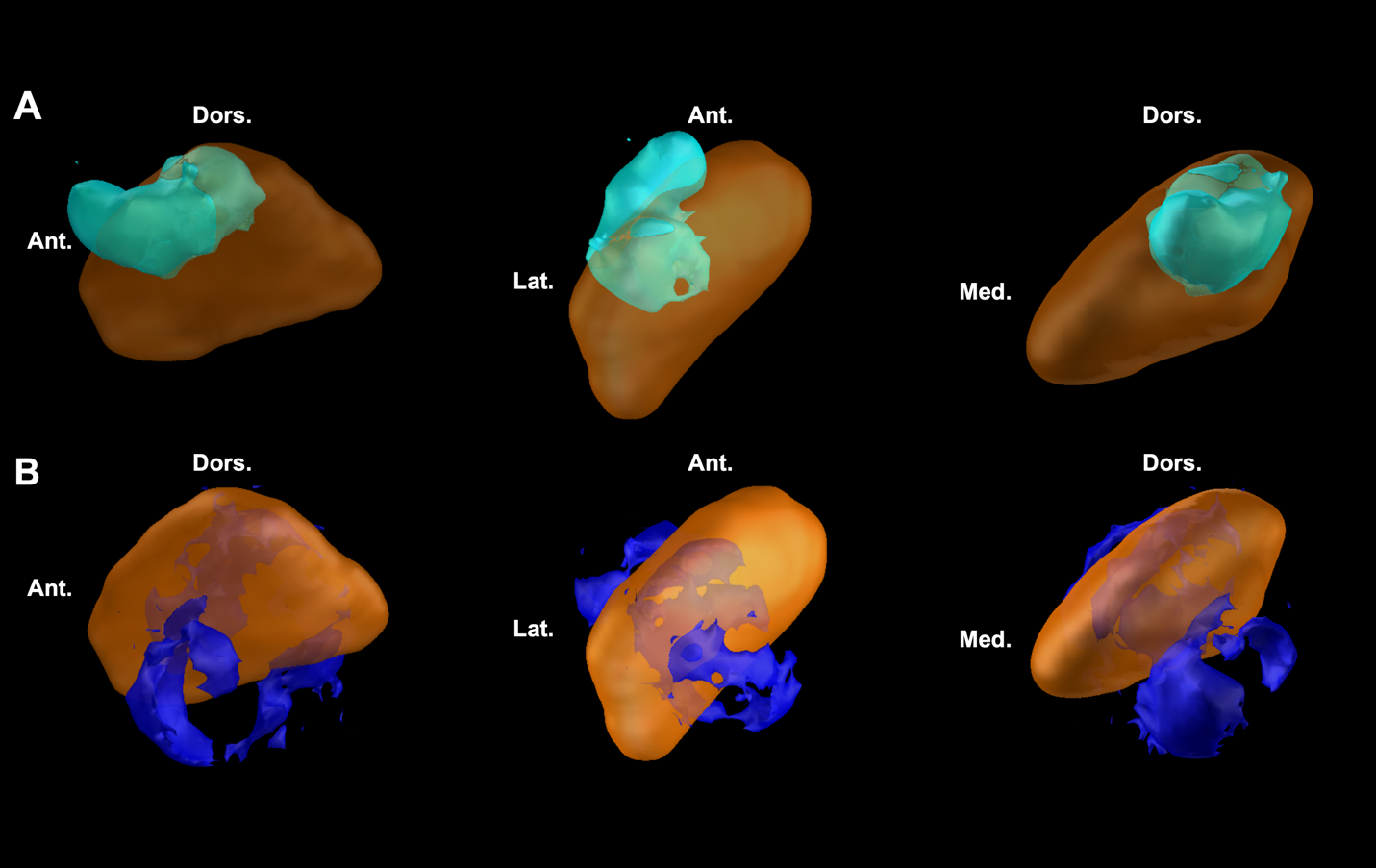


*Figure S1 A) A thresholded t-image based on ExecDeltas and DS VTAs is shown in cyan. This region indicates a volume where DS offers less or no benefit over OS. B) Among patients who improved with DS (ExecDelta > 0, n=10), we determined which voxels were spared by DS VTAs relative to OS. The resultant volume is shown in blue.*
